# Attitudes and behaviors regarding sun exposure in Japan compared to Europe and North America

**DOI:** 10.1111/1346-8138.17217

**Published:** 2024-05-03

**Authors:** A. Morita, H. W. Lim, T. Passeron, C. L. Goh, H. Y. Kang, F. Ly, J. Ocampo‐Candiani, S. Puig, S. Schalka, L. Wei, A. L. Demessant, C. Le Floc’h, D. Kerob, B. Dreno, J. Krutmann

**Affiliations:** ^1^ Department of Geriatric and Environmental Dermatology Nagoya City University Graduate School of Medical Sciences Nagoya Japan; ^2^ Department of Dermatology Henry Ford Health Detroit Michigan USA; ^3^ Department of Dermatology Côte d'Azur University, Nice University Hospital Center Nice France; ^4^ INSERM U1065, C3M Côte d'Azur University Nice France; ^5^ National Skin Centre Singapore Singapore; ^6^ Department of Dermatology University School of Medicine Suwon South Korea; ^7^ Department of Dermatology Cheikh Anta Diop Dakar University, EPS Institute of Social Hygiene Dakar Senegal; ^8^ Universidad Autonoma de Nuevo León, Facultad de Medicina University Hospital "Dr. Jose E. González" Monterrey Mexico; ^9^ Dermatology Department Hospital Clinic de Barcelona, Barcelona University Barcelona Spain; ^10^ Medecin Skin Research Center and Biochemistry Department Chemistry Institute of Sao Paulo University Sao Paulo Brazil; ^11^ Department of Dermatology The General Hospital of air Force PLA Beijing China; ^12^ La Roche‐Posay International Levallois‐Perret France; ^13^ Univ Angers, INSERM, Immunology and New Concepts in ImmunoTherapy, INCIT, UMR 1302 Nantes University Nantes France; ^14^ IUF Leibniz Research Institute for Environmental Medicine Duesseldorf Germany; ^15^ Medical Faculty Heinrich‐Heine‐University Duesseldorf Germany

**Keywords:** Japan, public health, sun exposure

## Abstract

The objective of our study was to assess the attitudes and behaviors in Japan regarding sun exposure and compare them to those in Europe and North America. The study population was a representative sample of individuals aged >18 years from Ipsos panels in Japan (*N* = 1000), North America (*N* = 1000), and Europe (*N* = 6000) using the quota method. Questionnaires covered habits, practices, and perceptions regarding sun exposure. Results revealed that the majority of people (80.1%) believed that the sun gives them energy, and 61.1% considered that being tanned made them look healthier. However, there was a significant difference between men and women regarding the appeal of tanned skin, with 54.95% of men versus 34.67% (*p* < 0.001) of women seeing a tan as an aesthetic asset. People aged <40 years were less likely to find a tan attractive (30.3%) compared to those aged ≥40 years (48.9%) (*p* < 0.001). Of those questioned, 45.70% of used sunscreen with a much higher use among women (70.10%) than men (18.74%) (*p* < 0.001). Almost 54% of people said they stayed in the shade to protect themselves from the sun with this behavior being more prevalent among women (67.05%) and fair‐skinned individuals (56.13%). Fear of the risks of sun exposure was more common among women, with 84.8% fearing premature skin aging, compared to 71.8% of men (*p* < 0.001). In Japan, 44.30% of those questioned said tanned skin was attractive (*p* < 0.001); for Europeans and North Americans the proportions were 81.1% and 77.6%, respectively. Only a quarter (25.80%) thought it essential to return from vacation with a tan. On the other hand, Europeans showed a strong recognition of the energy the sun brings (83.18%), and widely believed that tanned skin is attractive (82.32%) and healthy (73.15%). In North America, attitudes were similar to those in Europe regarding the attractiveness of tanned skin (77.65%) and the importance of returning tanned from vacation (48.15%). Compared to Europeans and North Americans, the Japanese seemed to be more cautious about sun‐induced hazards and considered lighter skin to be more attractive.

## 1 INTRODUCTION

Protection of the skin from sun exposure is increasingly being recognized as a major public health issue^1^ as knowledge about the risks of ultraviolet (UV) radiation is growing. It is well documented that UV radiation causes skin cancer, sunburn, premature aging, immunosuppression, and activation of latent viruses. While climate factors may influence the level of UV radiation at the earth's surface, it is individual behavior that has the greatest impact on exposure to UV radiation.^1^, ^2^ Data on attitudes to sun exposure in Japan are lacking. The objective of our study was to assess attitudes and behaviors regarding sun exposure in Japan and compare them to those in Europe and North America.

## 2 METHODS

The survey was conducted online from 28 September to 18 October 2021. The study population, selected from Ipsos online panels, included men and women aged ≥18 years from Japan (*N* = 1000), North America (USA & Canada, *N* = 1000), and Europe (France, Germany, Italy, Russia, Spain, and the UK *N* = 6000). Eligibility required that all participants had not recently taken part in a similar survey. A preliminary sample population was compiled using the automatic selection process of the Ipsos software (eMethodology). The preliminary sample was adjusted, giving a final sample population that fit the quotas^3^, ^4^ based on sex, age, employment status, and regions of the individual countries. The final sample size made allowances for country‐specific variations in response rates.

The questionnaires covered demographics, personal medical history, and sun‐exposure habits and practices. Information on phototypes (skin types) was documented using the Fitzpatrick classification, together with a description of the color of the skin and color picture representations. The questionnaires were translated into the appropriate languages for each country and proofread by a native speaker.

Data were analyzed using descriptive statistics, including frequency tables, means, standard deviations, and 95% confidence intervals. Two‐sided chi‐squared tests with a 0.05 significance level were used to compare subgroups. The Cosi software (M.L.I., France, 1994) was used for all analyses.

This survey was conducted in accordance with the ICC/ESOMAR code of conduct.

## 3 RESULTS

Table 1 shows the results of a survey of 1000 individuals in Japan on sun‐related perceptions, protection habits and their knowledge of the impact and risks of sun exposure, comparing responses by sex, age, and skin type.

**TABLE 1:** Attitudes and behaviors of the Japanese regarding sun exposure according to sex, age, and phototype.

	Global Japan population *n* = 1000		Men		Women		*p*	Under 40 years old		40 years and over		*p*‐Value	Fair skin		Dark skin		*p*‐Value
			*n* = 475		*n*= 525			*n* = 332		*n* = 668			*n* = 702		*n* = 298		
	*N*	%	*N*	%	*N*	%		*N*	%	*N*	%		*N*	%	*N*	%	
Preconceptions																	
Individual thinks/believes that…																	
*The sun gives us energy*	801	80.1	383	80.6	418	79.6	0.748	213	55.8	588	88.0	<0.001	558	79.5	243	81.5	<0.001
*Tanned skin is attractive*	443	44.3	261	54.9	182	34.7	<0.001	116	30.4	327	49.0	<0.001	294	41.9	149	50.0	0.472
*Being tanned makes us look healthier*	611	61.1	323	68.0	288	54.9	<0.001	154	40.3	457	68.4	<0.001	420	59.8	191	64.1	0.004
*It's essential to come back from holiday with a tan*	258	25.8	157	33.1	101	19.2	<0.001	64	16.8	194	29.0	<0.001	162	23.1	96	32.2	0.437
Sun protection habits																	
Individual is in the habit of…																	
*Using sunscreen*	457	45.7	89	18.7	368	70.1	<0.001	162	48.8	295	44.2	0.188	344	49.0	113	37.9	<0.001
*Wearing a cap or hat*	490	49.0	225	47.4	265	50.5	0.358	107	32.2	383	57.3	<0.001	330	47.0	160	53.7	0.159
*Wearing protective clothes*	355	35.5	108	22.7	247	47.0	<0.001	105	31.6	250	37.4	0.083	258	36.8	97	32.6	<0.001
*Wearing sunglasses*	190	19.0	93	19.6	97	18.5	0.716	45	13.6	145	21.7	0.003	134	19.1	56	18.8	0.094
*Staying in the shade*	539	53.9	187	39.4	352	67.0	<0.001	158	47.6	381	57.0	0.006	394	56.1	145	48.7	<0.001
Preparing to tan																	
Individual reports using																	
*Sunbeds*	51	5.1	34	7.2	17	3.2	0.008	24	7.2	27	4.0	0.045	35	5.0	16	5.4	0.685
*Gradual self‐tanners, cosmetics*	65	6.5	26	5.5	39	7.4	0.261	29	8.7	36	5.4	0.059	49	7.0	16	5.4	0.094
*Vitamin D supplements*	150	15.0	69	14.5	81	15.4	0.756	55	16.6	95	14.2	0.377	113	16.1	37	12.4	0.006
*Oral supplements (beta‐carotene, lycopene, lutein…)*	95	9.5	42	8.8	53	10.1	0.571	30	9.0	65	9.7	0.812	61	8.7	34	11.4	0.955
*Phototherapy under dermatological supervision*	31	3.1	10	2.1	21	4.0	0.123	16	4.8	15	2.2	0.044	23	3.3	8	2.7	0.369
*Hydroxychloroquine*	37	3.7	20	4.2	17	3.2	0.518	14	4.2	23	3.4	0.665	30	4.3	7	2.3	0.056
Following recommendations																	
Individual reports																	
*Avoiding the sun at the hottest part of the day (12 noon–4 pm)*	374	37.4	134	28.2	240	45.7	<0.001	119	35.8	255	38.2	0.517	280	39.9	94	31.5	<0.001
*Applying sunscreen (at least) every 2 h*	49	4.9	20	4.2	29	5.5	0.416	18	5.4	31	4.6	0.702	41	5.8	8	2.7	0.008
*Keep applying sunscreen even when tanned*	149	14.9	89	18.7	60	11.4	0.002	40	12.0	109	16.3	0.091	100	14.2	49	16.4	0.624
Individual knows that…																	
*The sun accelerates skin aging*.	785	78.5	354	74.5	431	82.1	0.005	193	58.1	592	88.6	<0.001	551	78.5	234	78.5	<0.001
*The sun can cause skin health problems*	701	70.1	323	68.0	378	72.0	0.190	177	53.3	524	78.4	<0.001	496	70.7	205	68.8	<0.001
Individual fears																	
*The risk of premature skin aging*	786	78.6	341	71.8	445	84.8	<0.001	202	52.9	584	87.4	<0.001	545	77.6	241	80.9	0.048
*The risk of developing cancer*	789	78.9	342	72.0	447	85.1	0.725	198	51.8	591	88.5	<0.001	550	78.3	239	80.2	0.018

The results revealed that the majority of people (80.1%) believed that the sun gives them energy, and 61.1% considered that being tanned made them look healthier. However, there was a significant difference between men and women regarding the appeal of tanned skin, with 54.9% of men versus 34.7% (*p* < 0.001) of women seeing it as an aesthetic asset. People <40 years were less likely to find a tan attractive (30.3%) compared to those aged ≥40 years (48.9%) (*p* < 0.001).

In terms of sun protection habits, 45.7% of people used sunscreen with much higher use among women (70.10%) than men (18.74%) (*p* < 0.001). It is also interesting to note that 53.9% of people said they stayed in the shade to protect themselves from the sun. This behavior was more prevalent among women (67.1%) and fair‐skinned individuals (56.1%).

Regarding the impact and risks of sun exposure, a large proportion of those questioned (78.5%) said they knew that the sun accelerates skin aging, and 70.1% recognized that the sun can cause skin health problems (*p* < 0.001). Fear of such risks was more common among women, with 84.8% fearing premature skin aging, compared to 71.8% of men (*p* < 0.001).

**FIGURE 1:**
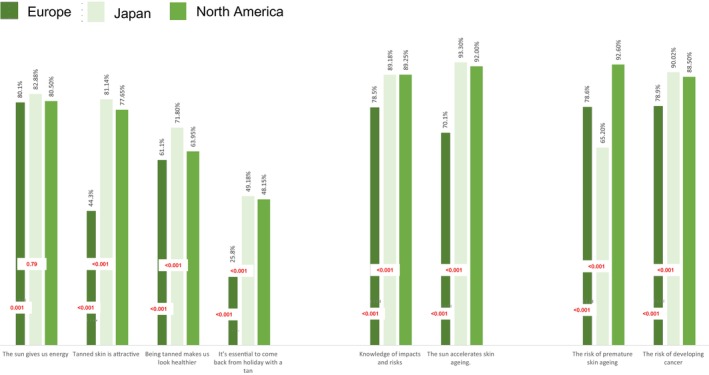
Comparison of perceptions of sun exposure in Japan, Europe, and North America. Percentages represent the proportion of individuals in the studied population of each region.

Figure 1 compares the perception and knowledge of the sun hazards among populations in Europe, Japan, and North America. Of those in Japan, 44.3% said they found tanned skin attractive, significantly lower (*p* < 0.001) than Europeans (81.1%) and North Americans (77.6%). Only a quarter of Japanese respondents (25.8%) thought it essential to return from vacation with a tan. On the other hand, Europeans showed a strong recognition of the energy the sun brings (83.2%), and widely believed that tanned skin is attractive (82.3%) and healthy (73.1%). It is also worthy of note that over half the Europeans (52.2%) thought it essential to return from vacation with a tan. In North America, attitudes were similar to those in Europe regarding the appeal of tanned skin (77.6%) and the importance of returning tanned from vacation (48.1%). They also showed a strong awareness of sun‐related risks, with 89.2% recognizing that the sun accelerates skin aging and 92% understanding that the sun causes skin health hazards.

## 4 DISCUSSION

This is the first study to assess the sun exposure habits and attitudes among the Japanese and compare them with such habits in Europe and North America. Overall, the results highlight significant differences in attitudes and behavior towards sun exposure, depending on sex, age, and skin type within the Japanese population. Compared to women, men considered that tanned skin is attractive, makes them look healthier, and is essential after a vacation. Women have a higher tendency to use sunscreen and stay in the shade. While women tend to avoid the sun at the peak hours of the day, men tend to apply sunscreen even after they are tanned. Individuals >40 years have more positive perceptions about the effect of the sun in their lives. However, they tend to wear a cap and sunglasses and stay in the shade more than those aged <40 years. Women and individuals aged >40 years expressed more fear of the risk of skin aging, but there was no difference between fair and dark‐skinned people. Those aged >40 years had more fear of the risk of skin cancer with no difference in regard to sex or skin type. While Europeans and North Americans share similar views on the appeal of tanning and the risks of the sun exposure, the Japanese show a more cautious attitude towards tanning. In fact, pale skin has historically been a standard of beauty in Asia, and until today, white skin represents beauty or wealth.^5^, ^6^ There exists a cultural preference for lighter and evenly toned skin in Asia, which may make Asians practice sun protection.^7^, ^8^ Previous studies have found that women raised in East Asian countries living in the USA express a desire for pale skin and, therefore, prefer to engage in activities out of the sun.^9^ On the other hand, white men and women tend to admire tanned skin, which can motivate skin cancer risk behaviors.^10^ One of the limitations in the study is the use of the Fitzpatrick classification. The classification, used for Japanese people, utilizes the Japan phototypes I, II and III. While it might sometimes be difficult to classify the phototype of Japanese people using only the Fitzpatrick classification, but the same classification had to be used on all individuals of the study.

## 5 CONCLUSION

In conclusion, sun protection habits and perception of skin hazards present in the Japanese population differ according to age, sex, and phototype. Compared to Europeans and North Americans, the Japanese seem to be more cautious regarding sun‐induced hazards and consider lighter skin to be more attractive. Given that sun exposure is a modifiable factor for skin cancer and photo‐ageing, promoting new cultural beliefs like those in Japan, and changing the definition of physical attractiveness might be a strategy to encourage sun‐smart behavior in campaigns against sun exposure. Culture and perception of tanned skin among the Japanese population seems to be a protective factor from sun exposure. With the increased use of social media, the promotion of the perception of light‐skin beauty on different online platforms could be considered to build a new trend in beauty alongside other beauty trends.

## CONFLICT OF INTEREST STATEMENT

Pr Thierry Passeron reports personal fees from La Roche Posay during the conduct of the study and personal fees from L'Oréal, ACM, Beiersdorf, Hyphen, ISDIN, ISIS Pharma, NAOS, SVR, SUN Pharma, Symrise. Pr. Henri W Lim is an investigator for Incyte, La Roche‐Posay, Pfizer and PCORI, has served as a consultant for ISDIN, Beiersdorf, Ferndale, L'Oreal, Eli Lily, and has been a speaker on general educational session for La Roche‐Posay, Cantabria labs, Pierre Fabre, NAOS, Uriage, and Pfizer. Pr. Chee Leok Goh reports other support from La Roche Posay during the conduct of the study. Pr. Kang reports personal fees from L'Oréal during the conduct of the study. Pr. Fatimata Ly has nothing to disclose. Pr. Morita reports personal fees from L'Oréal during the conduct of the study. Dr. Jorge Ocampo‐Candiani reports personal fees from La Roche Posay during the conduct of the study. Pr. Susana Puig reports personal fees from La Roche Posay, during the conduct of the study and personal fees and non‐financial support from La Roche Posay. Dr. Sergio Schalka has served as a consultant for La Roche Posay and has participated as a speaker in an educational session for La Roche‐Posay. Pr. Liu Wei reports personal fees from La Roche Posay during the conduct of the study. Anne‐Laure. Demessant‐Flavigny, Caroline Le Floc'h, Dr Delphine. Kerob are employees of La Roche Posay. Pr. Brigitte Dreno was investigator for BMS, Fabre, Novartis, Almirall, Regeneron, La Roche Posay, Bioderma. Pr. Jean Krutmann reports personal fees and grants from La Roche Posay during the conduct of the study and grants and personal fees from Amway, bitop, Blue Lagoon, Evonik, ISDIN, L'Oreal, Meitu, Mistine, Mibelle, RepliCell, Shin, Skinceuticals, Stada, Symrise, and Vichy.

## FUNDING INFORMATION

As part of the HELIOS project, this work was funded by La Roche Posay International.

## ETHICS STATEMENT

This survey was conducted according to the ICC/ESOMAR code of conduct.

## Data Availability

The data that support the findings of this study are available from the corresponding author upon reasonable request.
